# Emerging Multidrug-Resistant Hybrid Pathotype Shiga Toxin–Producing *Escherichia coli* O80 and Related Strains of Clonal Complex 165, Europe

**DOI:** 10.3201/eid2412.180272

**Published:** 2018-12

**Authors:** Aurélie Cointe, André Birgy, Patricia Mariani-Kurkdjian, Sandrine Liguori, Céline Courroux, Jorge Blanco, Sabine Delannoy, Patrick Fach, Estelle Loukiadis, Philippe Bidet, Stéphane Bonacorsi

**Affiliations:** Hôpital Robert-Debré (AP-HP),; Paris, France (A. Cointe, A. Birgy, P. Mariani-Kurkdjian, S. Liguori, C. Courroux, P. Bidet, S. Bonacorsi);; Université Paris Diderot, Sorbonne Paris Cité, Paris (A. Cointe, A. Birgy, P. Mariani-Kurkdjian, P. Bidet, S. Bonacorsi);; Universidade de Santiago de Compostela, Lugo, Spain (J. Blanco); ANSES, Plateforme IdentyPath, Maisons-Alfort, France (S. Delannoy, P. Fach);; Université de Lyon, CNRS, INRA, UCBL, VetAgro Sup, Laboratoire d’Écologie Microbienne, Villeurbanne, France (E. Loukiadis)

**Keywords:** Escherichia coli, Shiga toxin-producing E. coli, STEC, enterohemorrhagic E. coli, EHEC, hybrid pathotype, extra-intestinal virulence, emergence, antimicrobial resistance, multidrug resistance, foodborne diseases, whole-genome sequencing, clonal complex, CC165, Europe, food safety, enteric infections, bacteria

## Abstract

Enterohemorrhagic *Escherichia coli* serogroup O80, involved in hemolytic uremic syndrome associated with extraintestinal infections, has emerged in France. We obtained circularized sequences of the O80 strain RDEx444, responsible for hemolytic uremic syndrome with bacteremia, and noncircularized sequences of 35 O80 *E. coli* isolated from humans and animals in Europe with or without Shiga toxin genes. RDEx444 harbored a mosaic plasmid, pR444_A, combining extraintestinal virulence determinants and a multidrug resistance–encoding island. All strains belonged to clonal complex 165, which is distantly related to other major enterohemorrhagic *E. coli* lineages. All *stx*-positive strains contained *eae*-ξ, *ehxA*, and genes characteristic of pR444_A. Among *stx*-negative strains, 1 produced extended-spectrum β-lactamase, 1 harbored the colistin-resistance gene *mcr1*, and 2 possessed genes characteristic of enteropathogenic and pyelonephritis *E. coli*. Because O80–clonal complex 165 strains can integrate intestinal and extraintestinal virulence factors in combination with diverse drug-resistance genes, they constitute dangerous and versatile multidrug-resistant pathogens.

Enterohemorrhagic *Escherichia coli* (EHEC), a subset of Shiga toxin–producing *E. coli* (STEC), are major foodborne pathogens responsible for outbreaks and sporadic cases of gastrointestinal diseases ranging from simple diarrhea to hemorrhagic colitis, characterized by bloody diarrhea. The most serious complication, particularly in young children, is hemolytic uremic syndrome (HUS), defined by a combination of renal failure, thrombocytopenia, and hemolytic anemia (*1*). Post-STEC HUS is a major worldwide public health concern because it is the primary cause of acute renal failure in children (*1*). These clinical features result mainly from the action of the phage-encoded Shiga toxin (Stx), of which there are 2 types: Stx1, which has 3 subtypes, Stx1a, 1c, and 1d; and Stx2, which has 7 subtypes, Stx2a–g. In typical EHEC, adhesion to the intestinal epithelium is mediated by the locus of enterocyte effacement (LEE), a chromosomal pathogenicity island (PAI), shared with Enteropathogenic *E. coli* (EPEC) strains, which encodes a type III secretion system (T3SS), an adhesin called intimin, and its receptor Tir. Intimin, encoded by the *eae* gene, is a major virulence factor (VF) involved in the intimate attachment of typical EHEC to intestinal epithelium, causing characteristic attaching and effacing lesions. EHEC enterohemolysin (*ehxA*) is a pore-forming cytolysin carried by a plasmid involved in EHEC virulence. This plasmid, initially described as part of the O157 serogroup (pO157) (*2*), can carry 2 additional VFs, a catalase peroxidase, encoded by *katP*, and a serine protease, encoded by *espP*, which can cleave human coagulation factor V and might be involved in the development of hemorrhagic colitis (*3*).

Serogroup O157 is the predominant STEC serogroup worldwide, but non-O157 serogroups are increasingly associated with post-STEC HUS, and the unusual serogroup O80 is emerging in France and Europe. In 2016, O80 represented the second most frequent serogroup isolated in France, after serogroup O26 (*4*). This phenomenon is no longer restricted to France; strains of serotype O80:H2, all belonging to sequence type (ST) 301, have been identified in Spain (*5*), the Netherlands (*6*), and Switzerland (*7*).

This serogroup is unique for several reasons. First, it is always associated with multiple determinants of resistance (i.e., resistance to aminopenicillin, aminoglycoside, nalidixic acid, cotrimoxazole, tetracycline, or phenicols), whereas a resistance phenotype is uncommon among EHECs, which are generally fully susceptible to antibiotics, except for rare clones, such as the epidemic O104:H4 German clone carrying a *bla*_CTX-M-15_ gene (*8*). Furthermore, unusual extraintestinal infections have recently been described for this serogroup (*9*,*10*), such as bacteremia, whereas EHEC is generally known to be a strictly intestinal pathogen. A recent case in the Netherlands illustrates the potential extreme pathogenicity of this serogroup; a 16-month-old boy died from multiorgan failure and extensive cerebral thrombotic microangiopathy attributable to an O80 Stx2d-producing *E. coli* strain (*6*). Finally, O80 EHEC appears to be a hybrid pathotype that combines intestinal VFs (Shiga toxin [*stx*], intimin [*eae*], enterohemolysin [*ehxA*]) and extraintestinal VFs (aerobactin [*iucC*], salmochelin [*iroN*], an iron uptake protein encoded by *sitABCD*; serum resistance protein [*iss_p_*], a putative secretion system I [*etsC*], omptin [*ompTp*], hemolysin [*hlyF*], and 2 bacteriocins [*cia* and *cva*]), suggesting the presence of a pS88-like plasmid (*11*). pS88 is a ColV plasmid, a key determinant of extraintestinal pathogenic *E. coli* virulence in poultry and humans. This plasmid is involved in neonatal meningitis (*11*) and could explain the occurrence of extraintestinal dissemination in these EHEC infections. The recent diffusion in Europe, high potential extraintestinal pathogenicity, and multidrug resistance (MDR) of this hybrid pathotype led us to further characterize these strains, which might represent a major public health concern.

## Methods

We further characterized O80:H2 EHEC by fully sequencing a recent representative strain, called RDEx444. RDEx444 was responsible for a highly severe case of post-STEC HUS, complicated by bacteremia, in an 8-month-old infant in February 2016 in Bourg-en-Bresse, France. Initial symptoms were febrile diarrhea with signs of sepsis. Blood and stool cultures were positive for O80 EHEC, but urine cultures remained negative, suggesting gut translocation that led to bacteremia. The appearance of biologic signs of HUS with oliguria necessitated transfer to intensive care, several blood transfusions, and 5 days of peritoneal dialysis.

**Figure 1:**
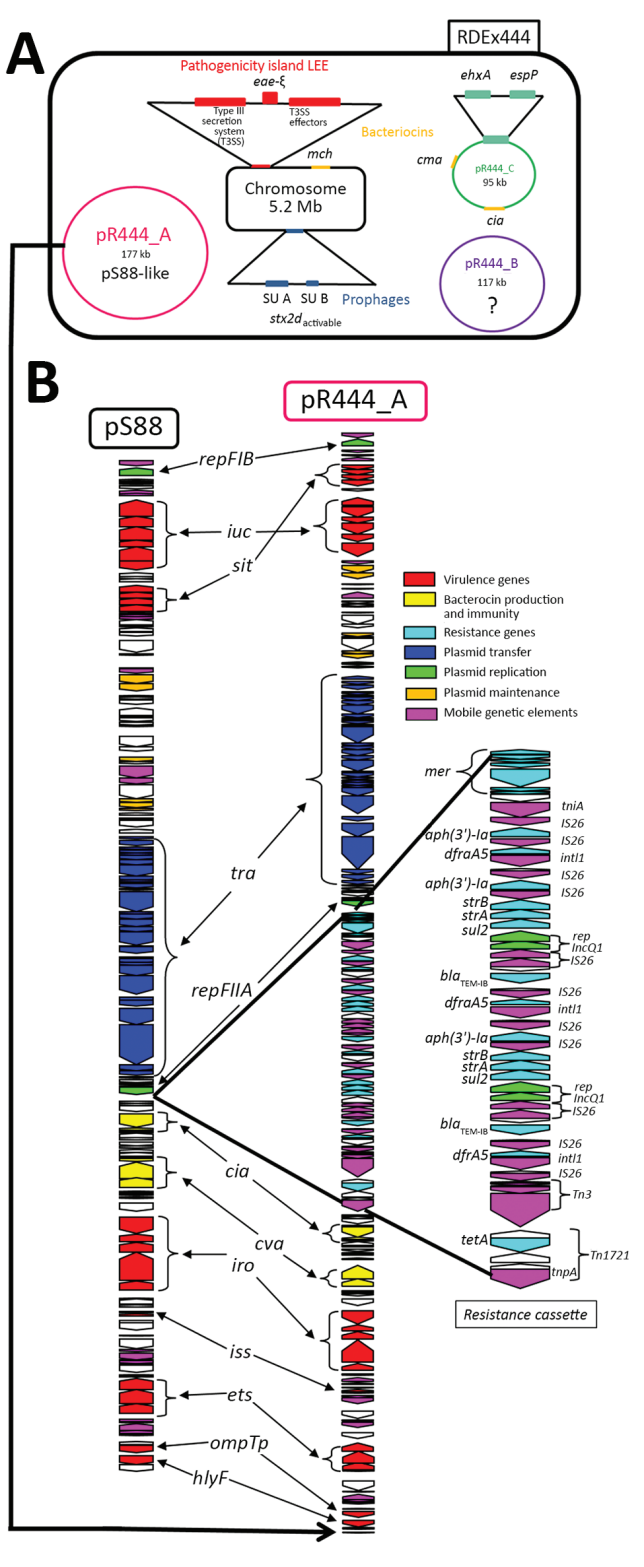
Genetic characterization of RDEx444, a strain of *Escherichia coli* serotype O80:H2 isolated in France in February 2016 and involved in hemolytic uremic syndrome with bacteremia, carrying both intestinal and extraintestinal virulence factors associated with (multidrug-resistance determinants (A) and genetic comparison between plasmid pS88 and mosaic plasmid pR444_A (B). A) Four circularized contigs (chromosome of 5,256,050 bp and the 3 plasmids pR444_A [176,500 bp], pR444_B [117,090 bp], and pR444_C [95,050 bp]) obtained by using PacBio (Pacific Biosciences, Meno Park, CA, USA) sequencing of RDEx444 are schematically represented. The main virulence factors are presented as colored rectangles. Intestinal virulence factors are indicated in red for the locus of enterocyte effacement genes, blue for prophage-encoded Shiga toxin genes, and green for VFs carried by pR444_C, a pO157-like plasmid. Bacteriocin genes (*mch, cia,* and *cma*) are indicated in yellow. B) Comparison of the sequences of pR444_A and pS88, the plasmid of strain S88 involved in neonatal meningitis. LEE, locus of enterocyte effacement.

We performed sequencing of RDEx444 by using the PacBio single-molecule real-time method with RS II chemistry 2.4.0 (Pacific Biosciences, Menlo Park, CA, USA); we used 1 single-molecule real-time cell. De novo assembly was performed twice by using the HGAP pipeline (https://github.com/PacificBiosciences/Bioinformatics-Training/wiki/HGAP) and annotation by using the MAGE platform (http://www.genoscope.cns.fr/agc/microscope/home/index.php) (Figure 1). For this strain, we performed plasmid typing by using databases (PlasmidFinder 1.3 and pMLST 1.4 [*12*]) available on the Center for Genomic Epidemiology (CGE) website (http://www.genomicepidemiology.org). Plasmids were also characterized by S1 nuclease pulsed-field gel electrophoresis (PFGE) with Southern hybridization and conjugability of the largest confirmed by experiments using rifampin-resistant *E. coli* J53, as previously described (*9*).

**Figure 2:**
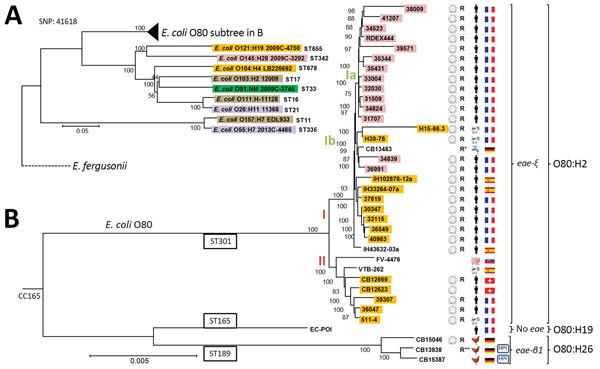
Phylogeny of 36 *Escherichia coli* serogroup O80 strains isolated from various sources and countries in Europe during 1998–2016 and their relationship to major enterohemorrhagic *E. coli* lineages. General phylogenic tree rooted on *E. fergusonii* (GenBank accession no. NC_011740), showing (A) the position of O80 strains among major enterohemorrhagic *E. coli* lineages (O157:H7 EDL933 NC_002655.2, O26:H11 11368 NC_013361.1, O111:H- 11128 NC_013364.1, O103:H2 12009 NC_013353.1, O55:H7 2013C-4465 CP015241, O91:NM 2009C-3745 JHGW00000000, O104:H4 LB226692 EO104H4LB.1, O145:H28 2009C-3292 JHHD00000000, and O121:H19 2009C-4750 JHGL00000000) and (B) a focused view of clonal complex 165, including O80 strains. The highlighted strains carry the Shiga toxin genes; the subtype of Stx is indicated by a color code as follows: purple, *stx1a*; yellow, *stx2a*; pink, *stx2d_activable_*; brown, *stx1a2a*; dark green, *stx1a2b*. The presence of the pS88 like–plasmid is represented by using a plasmid scheme next to the strain number. R to the right of the plasmid indicates that the strain possesses >2 resistance genes that confer resistance to β-lactams, kanamycin, or cotrimoxazole (R* indicates presence of additional extended-spectrum β-lactamase gene; R** indicates presence of *mcr-1* gene conferring additional resistance to colistin). The strain origin (country and source of isolation) is represented by flags and human, animal, or water symbols. CC, clonal complex; HPI, high-pathogenicity island (presence of chromosomal locus encoding the siderophore yersiniabactin); SNP, single-nucleotide polymorphism; ST, sequence type. Scale bar indicates nucleotide substitutions per site.

We also sequenced a representative set of 35 O80 strains from various sources and countries using the Nextera kit (Illumina, San Diego, California, USA) to prepare the library. Sequencing was performed by using a MiSeq reagent kit V3 600 cycles (Illumina). This panel included human isolates from France (n = 21), Spain (n = 3), and Switzerland (n = 2), as well as animal and environmental strains isolated in France (n = 3), Spain (n = 1), Slovakia (n = 1), and Germany (n = 4) (Figure 2). Some of the human strains from France and strains from Spain and Slovakia have been partially characterized previously (*10*). The strains from Spain and Slovakia were isolated during 1998–2012 and the strains from France during 2010–2016. The dates of isolation of the strains from Switzerland and Germany were not available. We performed assembly by using CLC Genomics Workbench software and SPAdes, also available on CGE website. The RAST server (http://rast.nmpdr.org) was used for genome annotation and the PHASTER web server (http://phaster.ca) to identify and annotate prophage sequences within bacterial genomes and plasmids. We established phylogeny by single-nucleotide polymorphism alignments between the contigs generated by CLC Genomics of O80 strains and 9 reference EHEC strain sequences of major serotypes available in GenBank (O157:H7 EDL933, O26:H11 11368, O111:H- 11128, O103:H2 12009, O55:H7 2013C-4465, O91:NM 2009C-3745, O104:H4 LB226692, O145:H28 2009C-3292, and O121:H19 2009C-4750 [Technical Appendix]) using CSIPhylogeny 1.4 (*13*). The neighbor-joining tree was built by using MEGA 3.1 (https://www.megasoftware.net) using bootstraps calculated from 100 replicates. The maximum-likelihood method yielded the same results (Technical Appendix Figure 1).

We performed multilocus sequence typing and identification of acquired antimicrobial resistance genes for all strains by using suitable databases available on the CGE website (SerotypeFinder 1.1 [*14*], MLST 1.8 [*15*], and ResFinder 3.0 [*16*]). Investigation of the resistome was completed by using the Resistance Gene Identifier at the Comprehensive Antibiotic Resistance Database website (https://card.mcmaster.ca/analyze/rgi). Moreover, 166 genes of intestinal and extraintestinal VFs (list available from authors) were assessed by local BLAST+ 2.2.31 analysis (https://blast.ncbi.nlm.nih.gov). Complete results are described in Technical Appendix Figure 2. Nucleotide sequences of the 36 sequenced O80 strains (complete nucleotide sequence of RDEx444 and 35 draft nucleotide sequences) have been deposited in Genbank (project no. PRJNA449634); accession numbers are available in the Technical Appendix.

## Results

RDEx444 can be considered to be representative of hybrid pathotype strains because it was responsible for an extraintestinal infection and showed similar virulence and resistance profiles as other strains described elsewhere (*9*,*10*). Complete sequencing yielded 2.3 × 10^9^ bp with 165,041 reads and an N50 (the length of the smallest contig among the set of the largest contigs that together cover >50% of the assembly) read length of 19,839 bp.

Four circularized contigs were obtained, including the chromosome of 5,256,050 bp, containing 5,146 coding sequences (CDSs), with an overall G+C content of 50.7% and 3 plasmids of 176,500 (pR444_A), 117,090 (pR444_B), and 95,050 bp (pR444_C). The number and size of the RDEx444 plasmids were corroborated by S1 nuclease PFGE (data not shown). We compiled a schematic representation of the genetic content, including major VFs, of this strain (Figure 1, panel A).

As expected, RDEx444 belongs to serotype O80:H2 and ST301. Among the 12 prophage regions identified on its chromosome, this strain carries a complete Stx converting bacteriophage of 43.9 kb. RDEx444 carries the *stx2d_activable_* variant, which has been shown to be predictive of severe clinical outcomes and progression to HUS (*17*). The Stx-prophage is integrated into the chromosomal *yecE* gene, a known integration site (*18*,*19*), initially reported in a strain producing Stx2e, encoded by the phage φP27 (*20*). The ≈15-kb region between Stx subunit B and the phage integrase, comprising proteins for DNA replication and repression, shares strong homology with the phage φP27. RDEx444 is otherwise a typical EHEC, given that it harbors a complete LEE and does not possess atypical adhesins, such as autoagglutinating adhesion (*saa*) or aggregative factors (*aggA/R*). RDEx444 carries the rare variant of the intimin gene *eae*-ξ (*5*), which has only very rarely been observed among other EHEC serotypes, but which is shared by all previously described O80 EHEC and EPEC isolates (*5*–*7*,*9*,*10*). Two other chromosomal traits of RDEx444 might also confer a selective advantage: 1) a region encoding microcin H47 (*mch A*, *S*, *X*, *B*, *C*, *D*, *E*, and *F*) ≈35 kb downstream from the Stx prophage, and 2) a chromosomal mutation in DNA gyrase (*gyrA S83L*), which confers quinolone resistance.

Plasmid pR444_C is 95,050 bp long with a G+C content of 49.8% and was predicted to contain 114 CDSs. Plasmid pR444_C is comparable to pO157 because it contains enterohemolysin (*ehxA*) and serine protease (*espP*) but does not possess catalase peroxidase (*katP*). Moreover, 2 other colicines, Ia (*cia*) and M (*cma*), are also present on this plasmid.

Plasmid pR444_B is 117,090 bp long with a G+C content of 46.5% and contains 135 CDSs and, unexpectedly, those for 3 tRNAs (threonine, asparagine, and tyrosine) redundant with those still present on the chromosome. This plasmid can be considered to be cryptic because it carries no drug resistance or virulence-associated genes, but several phage proteins are present. pR444_B shares high homology (99% nucleotide identity and 89% coverage) with plasmid pECOH89 (*21*), encoding an extended-spectrum β-lactamase *bla*_CTX-M-15_ identified in an *E. coli* strain and belonging to the family of phage-like plasmids. Members of this family are generally untypeable, nonconjugative, and cryptic plasmids, because no known virulence or resistance genes have been identified. Their function is unknown, but they all have strong homology to the *Salmonella*-specific bacteriophage SSU5 (*21*).

Plasmid pR444_A is 176,500 bp long, with a G+C content of 51.8%, and carries 2 replicons: FII_A and FIB_1 (ST [F2:A-:B1]). We identified 202 CDSs. This plasmid carries virulence-associated genes characteristic of pS88 (described previously) and MDR genes, and thus can be considered to be a mosaic plasmid. We identified an MDR-encoding region (48,237 bp) in addition to the plasmid-related function (56,106 bp) and virulence-associated domain (72,157 bp), closely related to pS88 (99% nucleotide identity and 96% query coverage) (*11*). This MDR-encoding region contains genes encoding resistance tetracycline (*tetA*), trimethoprim (*dfrA5*), sulfonamide (*sul2*), β-lactam (*bla*_TEM-IB_), kanamycin (*aph[3′]-Ia*), and streptomycin (*strA* and *strB*) and resistance against heavy metals such as mercury (*mer* gene) (Figure 1, panel B). This resistance cassette has only been described for pO26-CRL_125_ (100% nucleotide identity, 98% coverage) from an O26 EHEC but without VFs (*22*). Colocalization of extraintestinal VFs and MDR genes on the same plasmid was confirmed by Southern hybridization (data not shown). Moreover, we confirmed that this plasmid is conjugative, suggested by the presence on the annotation of an almost complete F-like transfer region, by successfully conjugating it with rifampin-resistant *E. coli* J53 (data not shown). Thus, this plasmid can diffuse by horizontal transfer.

Accordingly, this mosaic plasmid shows high homology with plasmid pS88, responsible for extraintestinal virulence in neonatal meningitis strains within which a resistance cassette has been integrated (Figure 1, panel B). Furthermore, pR444_A also carries 2 bacteriocin genes (*cia* and *cva*), such as pS88, which might confer a selective advantage by promoting intestinal establishment and colonization by killing other *E. coli* and freeing up their ecologic niches (Figure 1, panel B).

We sequenced a panel of strains consisting of representative O80 strains from France and all O80 strains from outside of France available at the beginning of the study to establish the genetic relationship between O80 strains isolated in several countries in Europe and analyze the diversity of their genetic content. We obtained an average of 233 contigs, with a mean depth of coverage of 58X and a mean N50 of 72,312. Statistics of each sequenced genome are summarized in Technical Appendix Table 1. Single-nucleotide polymorphism analysis (41,618 sites total) between the 36 sequenced O80 strains and 9 EHEC strains of other major serotypes (O157:H7, O26:H11, O111:H-, O103:H2, O55:H7, O91:NM, O104:H4, O145:H28, O121:H19) enabled us to establish a phylogenic tree (Figure 2), which shows 2 main clusters. EHEC O157:H7 (EDL933) and O55:H7 (2013C-4465) group together, as expected by their common origin, demonstrated by Feng et al. (*23*), and are distantly related to the other major EHEC serotypes, including the O80 strains. However, O80 isolates clearly form a separate group, suggesting that O80 emerged independently from the other serogroups. All of the O80 strains belong to clonal complex 165 (CC165), containing ST301, ST165, and ST189 (Figure 2, panel B).

Almost all of the O80 strains (32/36), including RDEx444, belong to the ST301 clonal group of serotype O80:H2. All of these isolates have EHEC markers, including genes encoded by the LEE, containing the rare variant of intimin *eae-ξ*, *ehxA*, and *stx2a* or *stx2d* genes, except for 4 strains missing the *stx* gene (CB13483, IH43632–03a, FV-4476, and VTB262) (Technical Appendix Figure 2). However, the presence of the other EHEC markers in these 4 strains (*eae*, *ehxA/espP,* or *katP*) suggests that they were initially STEC and underwent subsequent elimination of the *stx* gene. This finding led us to search for a scar of the Stx-converting bacteriophage at the insertion site described in the RDEx444 strain (*yecE*). We first searched for the contig carrying the *yecE* gene. Then, we used the Phaster webserver system to detect phage regions, which were finally blasted against RDEx444. We found scars of ≈31 kb in the human strain from Spain (IH43632-03a) and scars of ≈17 kb in the porcine strain from Slovakia (FV-4476), similar to the RDEx444 Stx-converting bacteriophage (92% and 94% nucleotide identity and 40% and 56% coverage, respectively) (data not shown). We found no scars for the other 2 strains, suggesting either complete prophage excision or insertion at another site.

All strains carrying the *stx* gene (28/36) also possess VFs typical of pS88, and all but 1 (CB12623) also carry genes conferring resistance to >1 of penicillin, tetracycline, kanamycin, or cotrimoxazole. The consistent association between extraintestinal VFs typical of pS88 and MDR genes, irrespective of the source or country of isolation, might suggest the presence of a mosaic plasmid, such as in the RDEx444 strain. Although it lacks pS88 markers, the water strain from Germany (CB13483) is nevertheless multidrug resistant and carries an extended spectrum β-lactamase gene (*bla*_CTX-M-1_), as well as the *mphA* gene, which confers resistance to azithromycin, the only antimicrobial drug that can be used for intestinal decontamination of EHEC (*24*; Technical Appendix Figure 2).

Among our panel, 3 STEC strains from cattle in France clearly belong to this ST301 clonal group. Cattle might thus represent an animal reservoir for these hybrid pathotype strains, given that the isolates possess exactly the same VFs (*eae*, genes of T3SS, *ehxA*, *stx*, and determinants of the pS88-like plasmid) and resistance genes as human EHECs.

Two clusters (I and II) can be distinguished within the ST301 group we describe. The main difference between these 2 groups concerns the presence of the unknown cryptic plasmid (pR444_B) in RDEx444. All strains of cluster I (n = 25) possess >85% of the genetic determinants of this plasmid of unknown function. BLAST results are depicted for each strain in Technical Appendix Table 2. Conversely, no strain of group II (n = 7), except 1, has this plasmid. No strains of ST165 or ST189 carry it either.

Within cluster I, the pS88-like plasmid carries 2 distinct gene profiles, showing its plasticity. The 12 Stx2d_activable_ EHECs isolated from humans in France (designated as subcluster Ia in Figure 2) carry the most complete form of the plasmid, identical to pS88. The aerobactin iron-uptake system, encoded by *iuc* genes, and the type I secretion system, encoded by *etsC*, are absent from the pS88-like plasmid of the subcluster Ib strains.

The second clonal group, ST165, is formed by 1 strain (EC_POI) of serotype O80:H19, which is devoid of all VF and resistance genes. This isolate might reflect the ancestral origin from which serogroup O80 EHEC strains were derived after the acquisition of diverse VFs.

Finally, clonal group ST189 consists of the 3 avian strains of serotype O80:H26 from Germany. None has *stx* or *ehxA* genes, but all have a complete LEE with the variant β1 of the intimin gene (*eae*). However, none of these strains carries the plasmid *bfp* gene of typical EPEC (Technical Appendix Figure 2). One strain (CB13938) has a region of ≈27 kb at the same insertion site (*yecE* gene) that shares homology with RDEx444 Stx-converting bacteriophage (88% nucleotide identity, 16% coverage), suggesting a potential scar of Stx-converting phage. Thus, this strain could have been originally an EHEC which secondarily lost its *stx* gene. No similar regions were found in the other 2 strains of this group. Otherwise, 1 strain (CB15046) has several VFs, confirming the presence of the pS88-like plasmid with MDR genes. The other 2 strains (CB15387 and CB13938) carry the locus encoding the siderophore yersiniabactin (*fyuA*), also called the high-pathogenicity island; these 2 strains also have pyelonephritis-associated pili with PapGII adhesin (Technical Appendix Figure 2). These strains constitute another type of hybrid pathotype with intestinal and extraintestinal VFs. Although none of them has VFs typical of pS88 plasmids, CB13938 is multidrug-resistant and carries the recently described *mcr-1* plasmid gene (*25*), conferring resistance to colistin. Blast analysis performed with the contig containing *mcr-1* (10.119 bp) shows strong homology (100% coverage, 99% identity) with 2 chromosomal insertion sites previously described in strains Mbl323 and Mbl506 (*26*).

## Discussion

We deciphered the molecular characteristics of O80:H2–CC165 EHEC, an emerging hybrid pathotype diffusing throughout Europe. This pathotype is armed to spread by means of a conjugative plasmid combining extraintestinal virulence with resistance to nearly all major classes of antibiotics, improved by the presence of several plasmid and chromosomally encoded bacteriocins, such as colicines I, V, M, and H47. We used the same criteria of MDR as a recent study in England (*27*) (*bla*_TEM-1_, *strA-strB*, *sul1/sul2/dfrA*, and *tetA*) and showed that 93% (26/28) of O80 STEC have this genotypic resistance profile, whereas only 5% of the strains identified within the O157 and O26 serogroups in the study in England had such a profile. MDR observed with this hybrid pathotype might complicate patient care, and the use of antimicrobial drugs during EHEC infections is still a subject of debate (*28*). However, the occurrence of invasive infections, such as bacteremia during EHEC infections, with this clone warrants antimicrobial treatment for such infections. In a previous study, the observed Stx rate was lower with a combination of azithromycin and ceftriaxone assays relative to basal secretion, and we proposed this association for the treatment of such infections (*10*).

Such a troubling plasmid has never been identified in human EHEC isolates. The only example of a similar mosaic plasmid was reported for *S. enterica* serovar Kentucky, in which an AmpC β-lactamase gene (*bla*_CMY-2_) was integrated into a pS88-like plasmid (*29*). The insertion of an MDR-encoding island in a pS88-like plasmid containing extraintestinal virulence genes is particularly worrisome. Massive and inappropriate use of veterinary antibiotics, such as tetracycline, in food-animal production promotes antimicrobial drug resistance among animals, known to be reservoirs for STEC. This practice can select and favor the spread of such MDR plasmids in human EHECs. Tetracycline still represented 36.5% of the tonnage of veterinary antibiotic use in 2015 in France (*30*). In our panel, all the sequenced O80 STEC strains carry the *tetA* gene, conferring resistance to tetracycline. Thus, large veterinary use of this drug might favor the selection of these hybrid strains and increase their diffusion.

We indicated a potential reservoir of these hybrid pathotype strains when we identified 3 O80:H2 strains isolated from cattle that carry the same VFs and resistance genes as human strains. However, the presence of the CC165 strains in chickens suggest that this clonal complex is also adapted to poultry. An initial description of pS88-like plasmids in avian pathogenic *E. coli* strains reinforces this hypothesis (*31*). Moreover, the environmental survival of this clone in these potential reservoirs might be enhanced because of the resistance to mercury shared by all but 1 strain (36047), all carriers of the pS88-like plasmid, irrespective of their origin. Such resistance to heavy metals has been rarely described in EHEC strains (*22*).

We also detected an O80:H19–CC165 strain devoid of virulence genes, which might represent the ancestral precursor of CC165, and from which these hybrid pathotype strains might have been derived. This strain could be used for tracing the genetic history of this clone in future studies.

Our genetic description of the emerging hybrid pathotype *E. coli* O80:H2, associated with O80-related strains, reveals the outstanding capacity of O80–CC165 to acquire the combination of virulence genes involved in intestinal and extraintestinal pathogenicity and genes conferring broad antibiotic resistance, including extended-spectrum β-lactamase–encoding genes and those most recently identified, such as *mcr-1*. O80–CC165 strains, which are able to integrate multiple VFs with various consequences, MDR genes that encompass nearly all classes, and bacteriocins, represent a serious threat because of their exceptional versatility and should therefore be closely monitored in all countries in Europe.

Technical Appendix Summary of sequencing data, GenBank accession numbers, phylogenetic analysis, technical data, and exhaustive list of genes identified by sequencing during study of *Escherichia coli* serogroup O80 strains in Europe.
