# Supplementary material for: Emerging Multidrug-Resistant Hybrid Pathotype Shiga Toxin–Producing Escherichia coli O80 and Related Strains of Clonal Complex 165, Europe
Source: Emerg Infect Dis. 2018 Dec;24(12):2262–9. doi: 10.3201/eid2412.180272 (PMC6256387; doi:10.3201/eid2412.180272)
Supplement: Technical Appendix — Summary of sequencing data, GenBank accession numbers, phylogenetic analysis, technical data, and exhaustive list of genes identified by sequencing during study of Escherichia coli serogroup O80 strains in Europe. [file 18-0272-Techapp-s1.pdf]

Article DOI: https://doi.org/10.3201/eid2412.180272

Emerging Multidrug-Resistant Hybrid Pathotype Shiga Toxin–Producing *Escherichia coli* O80 and Related Strains of Clonal Complex 165, Europe

Technical Appendix

GenBank Accession Numbers

We established phylogeny by single-nucleotide polymorphism alignments between the contigs generated by CLC Genomics of O80 strains and 9 reference EHEC strain sequences of major serotypes available in GenBank: O157:H7 EDL933 (accession no. NC_002655.2), O26:H11 11368 (NC_013361.1), O111:H- 11128 (NC_013364.1), O103:H2 12009 (NC_013353.1), O55:H7 2013C-4465 (CP015241), O91:NM 2009C-3745 (JHGW00000000), O104:H4 LB226692 (EO104H4LB.1), O145:H28 2009C-3292 (JHHD00000000), and O121:H19 2009C-4750 (JHGL00000000).

Nucleotide sequences of the 36 sequenced O80 strains (complete nucleotide sequence of RDEx444 and 35 draft nucleotide sequences) have been deposited in Genbank: Project PRJNA449634 under accession numbers: QBCG00000000, QBCH00000000, QBCI00000000, QBCJ00000000,QBCK00000000, QBCL00000000, QBCM00000000, QBCN00000000, QBCO00000000,QBCP00000000, QBCQ00000000, QBCR00000000, QBCS00000000, QBCT00000000, QBCU00000000, QBCV00000000, QBCW00000000, QBCX00000000, QBCY00000000, QBCZ00000000, QBDA00000000, QBDB00000000, QBDC00000000, QBDD00000000, QBDE00000000, QBDF00000000, QBDG00000000, QBDH00000000, QBDI00000000, QBDJ00000000, QBDK00000000, QBDL00000000, QBDM00000000, QBDN00000000, QBDO00000000, QBDP00000000.

Technical Appendix Table 1. Statistic of sequenced genomes

Strain number	No. of contigs	N50	No. of bases	Coverage (X)
38009	202	106 974	5 426 079	147.6
41207	211	107 035	5 467 265	79.4
34523	265	55 705	5 426 710	32.6
39571	228	84 038	5 476 022	119.3
35344	203	83 962	5 343 804	70.2
35431	269	43 457	5 314 749	31.8
33004	246	56 663	5 401 414	37.9
32030	237	67 378	5 486 203	43
31509	186	83 971	5 379 976	53.1
34824	281	39 286	5 366 586	31.3
31707	413	49 584	5 380 230	39.6
H15–66.3	185	121 183	5 457 940	123.9
H39–78	216	102 553	5 449 344	60.3
CB13483	250	45 176	5 302 150	37.4
34839	192	99 988	5 433 192	100.1
36991	292	38 410	5 457 973	31.4
IH102878–12a	197	88 042	5 554 738	67
IH33264–07a	242	49 429	5 277 481	41.3
37619	253	52 463	5 308 653	35.6
30347	194	84 601	5 358 355	48.1
33115	259	49 396	5 343 896	36.8
36549	206	71 156	5 375 868	59.9
40963	383	33 063	5 468 507	35.2
IH43632–03a	189	77 642	5 252 308	48.5
FV-4476	166	75 655	5 224 753	72.1
VTB-262	126	102 083	5 005 084	72.1
CB12669	191	88 417	5 317 409	65.4
CB12623	302	33 724	5 223 037	37.8
39307	176	102 609	5 503 620	106.7
36047	284	37 908	5 263 027	37.7
511–4	232	85 373	5 443 854	57.2
EC-POI	56	183 356	4 744 330	62.7
CB15046	290	35 034	5 225 546	34.8
CB13938	319	38 745	5 310 718	44.2
CB15387	215	56 890	5 169 961	33.8

Technical Appendix Table 2. Distribution of sequences homologous to the pR444_B plasmid among 08O:H2 hybrid STEC strains

Strain number	Contig number	Max score	Total score	Query cover	Identity
38009	91	91650	91789	42%	99%
	30	73706	1.091e+05	50%	100%
41207	19	91650	91789	42%	99%
	10	73719	1.091e+05	50%	99%
34523	99	73582	1.089e+05	50%	100%
	100	64467	64537	29%	99%
	214	26325	26394	12%	99%
39571	66	56604	56604	26%	99%
	45	49125	49195	22%	99%
	85	42492	45301	20%	99%
	40	35349	52330	24%	99%
35344	6	73584	1.089e+05	50%	100%
	34	54010	54079	25%	99%
	7	15254	17994	8%	99%
	26	12213	12282	5%	100%
35431	66	73582	1.089e+05	50%	100%
	48	53963	54033	25%	99%
	151	15254	17990	8%	99%
	166	12213	12282	5%	100%
33004	67	73656	1.090e+05	50%	99%
	115	65237	65467	30%	99%
	149	26256	26326	12%	99%
32030	44	91580	2.033e+05	93%	99%
31509	101	91613	94490	43%	99%
	24	73582	1.089e+05	50%	100%
34824	5	73582	1.089e+05	50%	100%
	47	64247	67055	31%	99%
	121	23736	23805	11%	99%
31707	36	73577	1.089e+05	50%	99%
	37	64379	67186	31%	99%
	117	26478	26547	12%	99%
H15–66–3	29	91563	2.033e+05	93%	99%
H39–78	42	91563	2.033e+05	93%	99%
CB13483	44	73538	2.027e+05	93%	99%
34839	4	91569	2.033e+05	93%	99%
36991	64	73538	1.536e+05	71%	99%
	49	26299	49982	23%	99%
IH102878–12a	7	73538	2.033e+05	93%	99%
IH-33264–07a	77	54769	1.270e+05	58%	99%
	124	36808	76261	35%	99%
37619	13	65442	1.049e+05	48%	99%
	109	35318	88540	40%	99%
30347	30	54894	94314	43%	99%
	52	32221	33229	15%	99%
	106	26559	26559	12%	99%
	101	26380	29564	13%	99%
	146	9858	9858	4%	100%
33115	29	66279	1.027e+05	47%	99%
	59	36684	64757	29%	99%
	111	26559	26559	12%	99%
36549	41	36662	64735	29%	99%
	40	35323	68797	31%	99%
	99	33883	33883	15%	99%
	21	26559	26559	12%	99%
40963	58	36684	64757	29%	99%
	98	35323	68747	31%	99%
	78	33883	33883	15%	99%
	200	26559	26559	12%	99%
IH43632–03a	32	51182	2.069e+05	95%	99%
FV-4476	114	4678	4678	2%	100%
	73	3613	3880	1%	99%
	22	3164	4103	2%	94%
	8	2106	2106	1%	91%
VTB-262	65	4676	4676	2%	100%
	44	3965	3965	2%	95%
	27	3158	4098	2%	94%
	32	2106	2106	1%	91%
	30	1256	2765	1%	99%

Strain number	Contig number	Max score	Total score	Query cover	Identity
CB12669	11	4676	4676	2%	100%
	44	3965	3965	2%	95%
	128	3531	3531	1%	99%
	43	3164	4103	2%	94%
	119	1256	2765	1%	99%
CB12623	30	4676	4676	2%	100%
	61	3164	4103	2%	94%
39307	23	57483	1.705e+05	83%	98%
	5	4676	4676	2%	100%
36047	244	4676	4676	2%	100%
	174	3164	4103	2%	94%
	15	2372	2372	1%	96%
	50	2100	2100	1%	91%
511–4	129	4676	4676	2%	100%
	76	3164	4103	2%	94%
	14	2372	2372	1%	96%
	149	2106	2106	1%	91%
	7	1256	2138	1%	99%
Ec-POI	6	3169	4258	2%	94%
CB15046	221	4340	4340	2%	98%
	64	3609	3609	1%	100%
	55	3164	4103	2%	94%
	190	2309	2309	1%	97%
CB13938	41	4617	4617	2%	100%
	165	4340	4340	2%	98%
	40	3164	4103	2%	94%
	73	2817	2817	1%	100%
CB15387	93	4340	4340	2%	98%
	65	3609	3609	1%	100%
	40	3164	4103	2%	94%

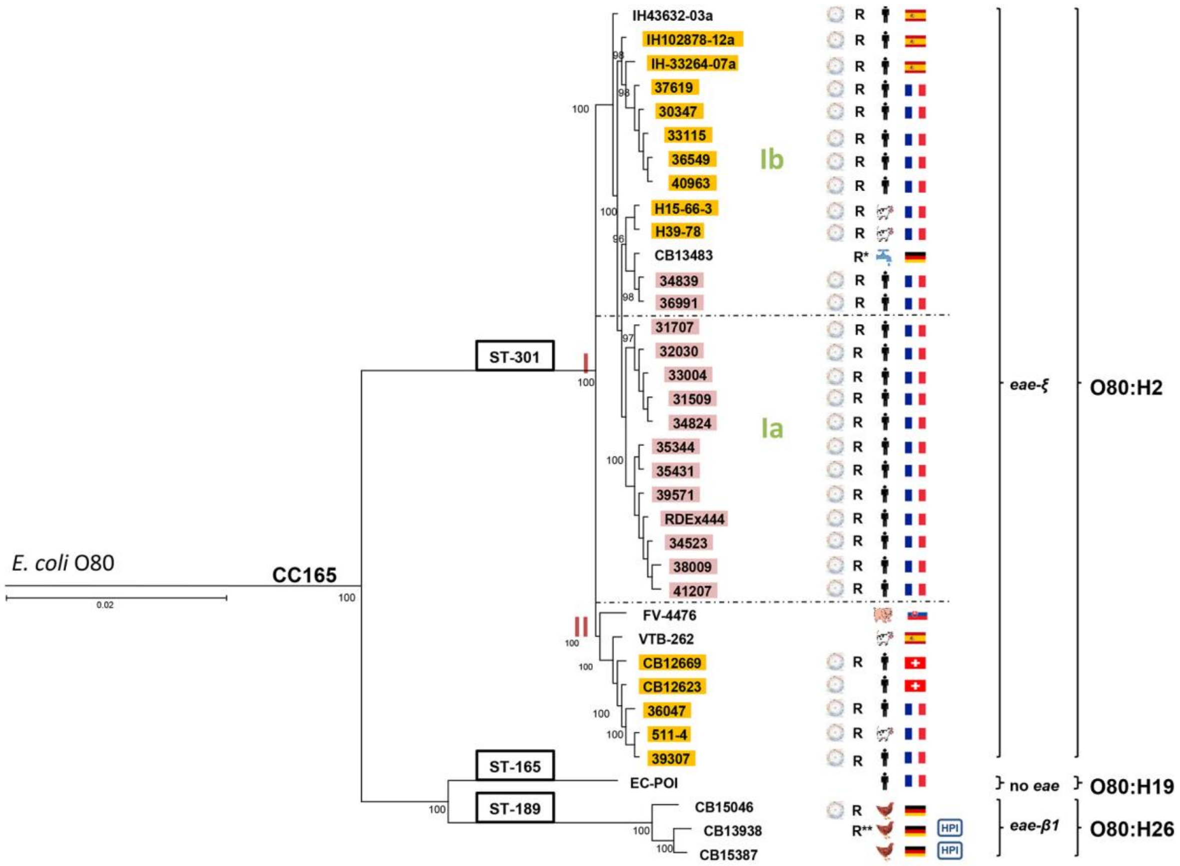

Technical Appendix Figure 1. Phylogeny (maximum likelihood method) of 36 O80 *E. coli* isolated from various sources and European countries between 1998 and 2016. The highlighted strains carry the Shiga toxin genes and the subtype of Stx is indicated by a color code as follows: purple, *stx1a*; yellow, *stx2a*; pink, $stx2d_{activable}$; brown, *stx1a2a*; dark green, *stx1a2b*. The presence of the pS88 like-plasmid is represented using a plasmid scheme next to the strain number. The "R" to the right of the plasmid indicates that the strain possesses at least two resistance genes that confer resistance to betalactams, kanamycin, or cotrimoxazole. R*: additional extended spectrum betalactamase (ESBL) gene. R**: additional resistance to colistin (*mcr-1* gene). The strain origin (country and source of isolation) is represented by flags and human/animal/water symbols. HPI: presence of chromosomal locus encoding the siderophore yersiniabactin, also called the High Pathogenicity Island.

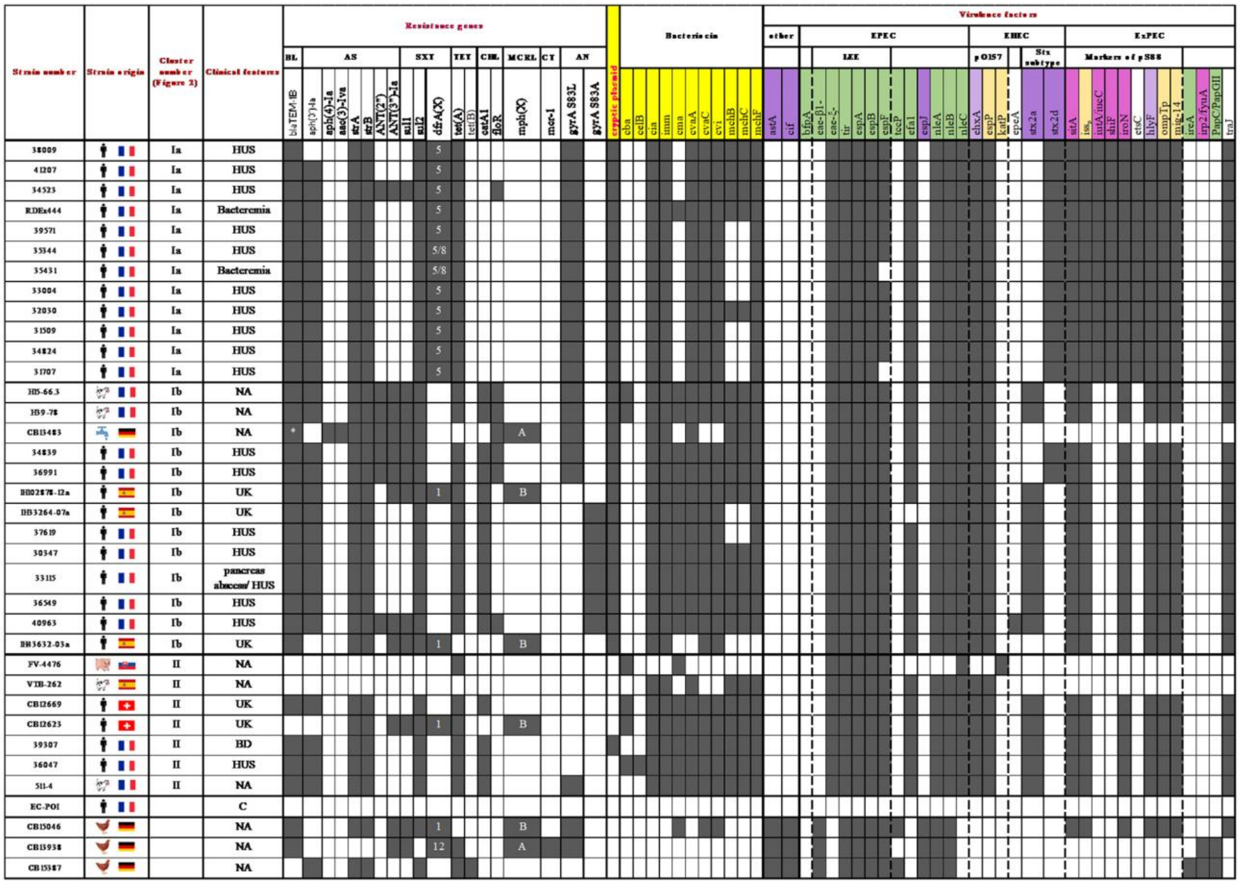

Technical Appendix Figure 2. Summary of complete sequencing data of the 36 O80 strains isolated in various European countries between 1998 and 2016. The strain origin (country and source of isolation) is represented by flags and human/animal/water symbols as in Figure 2. Clinical features are indicated as follows: HUS for hemolytic uremic syndrome, BD for bloody diarrhea, NA for not applicable (animal strains), UK for unknown, and C for healthy carrier. Gray boxes indicate the presence of resistance genes, virulence genes, or the presence of cryptic plasmids similar to pR444_B. Resistance genes are classified by antibiotic family: BL for betalactams, AS for aminoglycoside, SXT for cotrimoxazole, TET for tetracycline, CHL for phenicols, MCRL for macrolide, CT for colistin, and AN for nalidixic acid and numbers indicate the variant of the resistance genes. *variant *bla*$_{CTX-M-1}$ of extended spectrum betalactamase (ESBL) gene. Bacteriocins are indicated in yellow and the type of virulence factor is indicated by a color code as follows: purple, toxin; green, adhesion factor; light purple, hemolysin; beige, protectin; pink, iron uptake system.
